# MR-based radiomics-clinical nomogram in epithelial ovarian tumor prognosis prediction: tumor body texture analysis across various acquisition protocols

**DOI:** 10.1186/s13048-021-00941-7

**Published:** 2022-01-12

**Authors:** Tianping Wang, Haijie Wang, Yida Wang, Xuefen Liu, Lei Ling, Guofu Zhang, Guang Yang, He Zhang

**Affiliations:** 1 Department of Radiology, Obstetrics and Gynecology Hospital, Fudan University, Shanghai, China; 2 Shanghai Key Laboratory of Magnetic Resonance, East China Normal University, Shanghai, China

**Keywords:** Epithelial ovarian cancer, Radiomics, MR images, Prognosis, Computer-aided diagnosis

## Abstract

**Background:**

Epithelial ovarian cancer (EOC) is the most malignant gynecological tumor in women. This study aimed to construct and compare radiomics-clinical nomograms based on MR images in EOC prognosis prediction.

**Methods:**

A total of 186 patients with pathologically proven EOC were enrolled and randomly divided into a training cohort (*n* = 130) and a validation cohort (*n* = 56). Clinical characteristics of each patient were retrieved from the hospital information system. A total of 1116 radiomics features were extracted from tumor body on T2-weighted imaging (T2WI), T1-weighted imaging (T1WI), diffusion weighted imaging (DWI) and contrast-enhanced T1-weighted imaging (CE-T1WI). Paired sequence signatures were constructed, selected and trained to build a prognosis prediction model. Radiomic-clinical nomogram was constructed based on multivariate logistic regression analysis with radiomics score and clinical features. The predictive performance was evaluated by receiver operating characteristic curve (ROC) analysis, decision curve analysis (DCA) and calibration curve.

**Results:**

The T2WI radiomic-clinical nomogram achieved a favorable prediction performance in the training and validation cohort with an area under ROC curve (AUC) of 0.866 and 0.818, respectively. The DCA showed that the T2WI radiomic-clinical nomogram was better than other models with a greater clinical net benefit.

**Conclusion:**

MR-based radiomics analysis showed the high accuracy in prognostic estimation of EOC patients and could help to predict therapeutic outcome before treatment.

**Supplementary Information:**

The online version contains supplementary material available at 10.1186/s13048-021-00941-7.

## Introduction

Epithelial ovarian cancer (EOC) is the most malignant gynecological tumor in women [1]. The standard treatment is combined chemotherapy with carboplatin and paclitaxel after debulking surgery. However, most cases will relapse within 3 years after the first complete treatment cycle [2, 3]. Most of the patients who relapsed in half a year showed refractory chemotherapy resistance and had a poor prognosis [4, 5]. Therefore, how to select these patients as early as possible may help to design individualized treatment strategies (such as targeted immunotherapy) and improve the potential treatment outcome.

Magnetic resonance (MR) imaging is a method to evaluate the diagnosis of uncertain adnexal masses in ultrasound examination, which has high accuracy in the detection of malignant tumors [6–9]. In recent years, MR-based imaging informatics has been rapidly developed, which provides useful information for the classification of ovarian masses [10–13]. However, studies using preoperative radiologic images to predict therapeutic outcomes are limited [12, 14]. In one study, the authors used deep learning methods to extract computer tomography (CT) image features and reported the effective 3-year recurrence probability prediction from two institutions [15]. In our previous study, we found that the radiomic features of T1WI on the maximum lesion plane were most likely related to the clinical outcome [12].

Radiomics is an advanced tool for assessing tumor heterogeneity by analyzing medical images [16–18]. Its essence is to extract high-throughput quantitative features from high-quality medical images and establish a predictive model for diagnosis and prognostic evaluation [19–23]. Previous studies have reported that radiomics has potential in the classification of ovarian cystadenomas and stratification of ovarian cysts [24, 25]. A CT-based radiomics study has demonstrated the feasibility of predicting the risk of postoperative recurrence of advanced high-grade serous ovarian cancer [26].

Theoretically, MR has better soft tissue resolution and can provide more detailed tumor anatomy and biological information than CT. The purpose of this study is twofold: firstly, we compared the correlation between preoperative MR-based radiomic features and clinical outcomes in a large cohort sample; secondly, we evaluated the best predictor of MRI features (imaging biomarker) and compared its performance in different acquisition sequences.

## Materials and methods

### Patients selection

Our institutional review board (Obstetrics and Gynecology Hospital of Fudan University, Shanghai, China) approved this retrospective study, and the requirement for informed consent was waived for all participants. From January 2013 to December 2018, consecutive patients with clinically suspected gynecological diseases were retrospectively retrieved from our institutional Picture Archiving and Communication System (PACS, GE). The inclusion criteria were as follows: 1) no previous pelvic surgery; 2) no previous history of gynecological diseases; and 3) The MR examination before laparotomy or laparoscopic surgery was performed at our institution. The exclusion criteria were as follows: 1) previous pelvic surgery or radiotherapy; 2) MR imaging data were from outer institutions; and 3) no final pathological results or metastatic tumors. Finally, a total of 186 patients were included (mean age, 47.7 ± 13.2 years). The sample consisted of 55 patients with borderline tumors, 23 patients with clear cell tumors, 12 patients with endometrioid tumors, 9 with low grade tumors and 87 patients with high grade serous cancer. All included patients were pathologically confirmed by invasive surgery (laparoscopy or laparotomy). FIGO staging, pathological types, immunohistochemical staining results and laboratory examinations were collected through the hospital information system (HIS).

### Patients follow-up

All patients were followed up every 6 months for the first 3 years, and then annually thereafter. We used disease-free survival as the end point. The time range was defined as the number of days between the first day of treatment and the date of disease progression (determined by imaging or clinical examination), death, or the date of last follow-up survey. All the information was provided by the patient herself or her relative who knew the medical history.

### MR acquisition and lesion segmentation

MRI was performed using a 1.5 T MR system (Magnetom Avanto, Siemens) with a phased-array coil. Routine MRI protocols used for the assessment of pelvic masses included axial turbo spin-echo (TSE) T1-weighted imaging (T1WI), sagittal TSE T2-weighted imaging (T2WI), and axial/sagittal TSE fat-suppressed T2WI (fs-T2WI). Detailed MRI acquisition parameters are listed in supplementary Table 1. Diffusion weighted imaging (DWI) using a two-dimensional sequence of echo-planar imaging, performed in the axial plane with parallel acquisition technique by using b value = 0, 100, and 800 s/mm^2^. Pelvic enhanced imaging was acquired at multiple enhancement phases in sagittal and axial planes. All lesion segmentation was performed by an experienced radiologist (T.W.). We segmented all visible lesions on each slice on T1WI, T2WI, DWI and CE T1WI. For lesions with a wide range of peritoneal implants, we chose the largest part of the lesion. Itk-Snap software was used for volume of interest (VOI) segmentation [27].

### Radiomic feature extraction

**Fig. 1:**
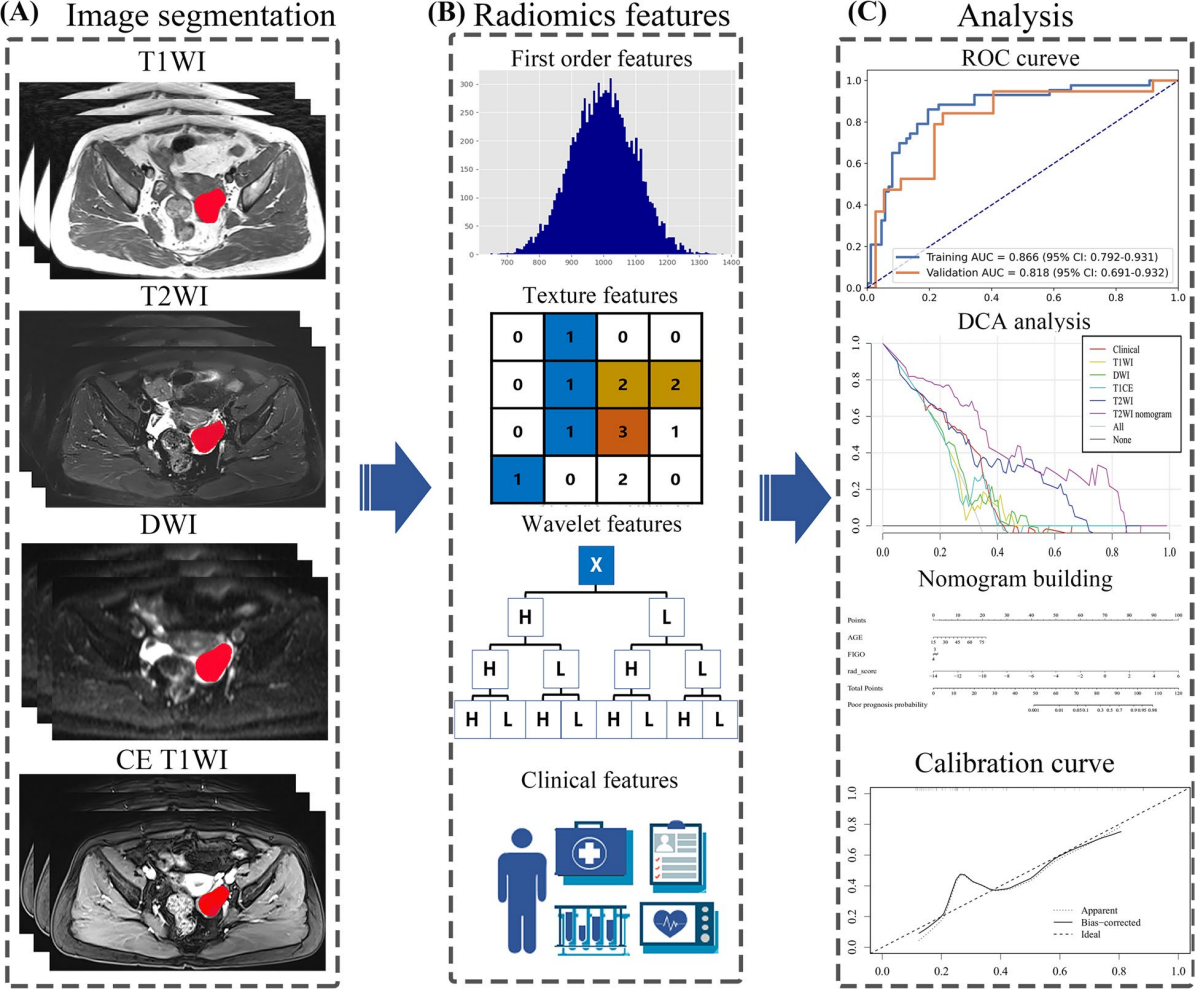
The flowchart of this study. The flowchart consists of three steps: **A** volume of interest manual segmentation, **B** Radiomics features extraction, **C** signatures and nomograms construction

The flowchart of this study was illustrated in Fig. 1. MR images of each sequence were collected from the same scanner with the same resolution. Feature extraction was performed using PyRadiomics (version 3.0.1, https://pyradiomics.readthedocs.io/) package for Python (version 3.8) [28]. Laplacian-of-Gaussian (LoG) filters with different λ-parameters (λ = 1.0, 3.0, 5.0) and wavelet filters were used for pre-processing the original T1WI, T2WI, DWI and CE T1WI images. A total of 1116 features were extracted from MR images of each patient. The radiomics features included: (1) 18 first-order statistics features; (2) 75 texture features including grey level co-occurrence matrix (GLCM), grey-level dependence matrix (GLDM), grey-level run-length matrix (GLRLM), grey-level size zone matrix (GLSZM) and neighborhood grey-tone difference matrix (NGTDM); (3) 279 statistical features derived from LoG filtered domain; (4) 744 wavelet features derived from wavelet filtered domains.

### Dataset split

**Fig. 2:**
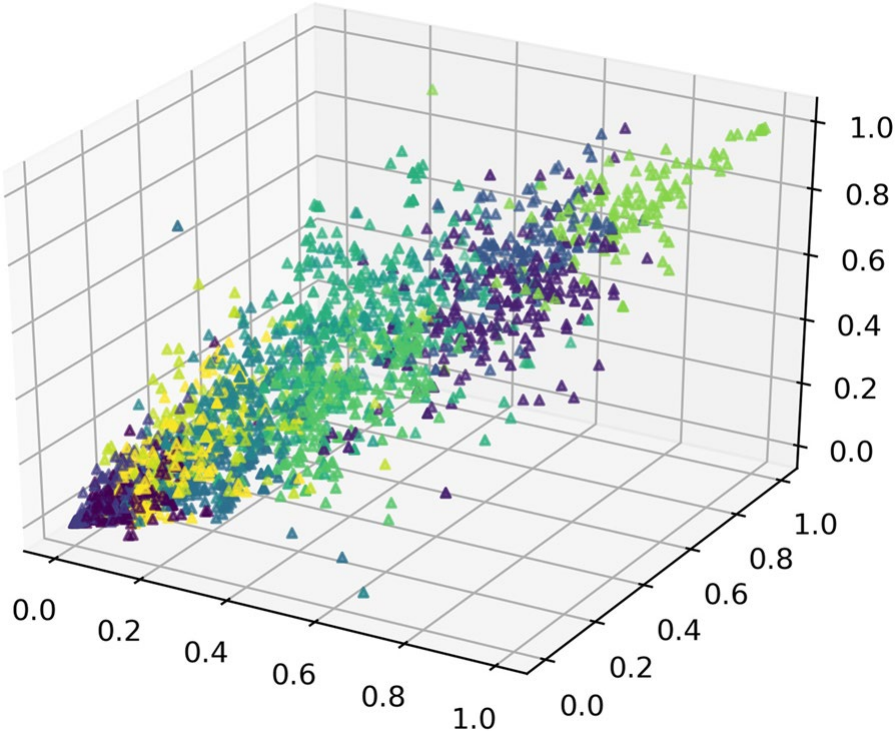
The three-dimensional (3D) visualization of the clustering result of all radiomics features. Different dots represent the individual projection of each radiomics feature in the 3D direction; the same color dots were assigned into one kind of cluster by K-means algorithm

Due to different distribution of radiomics features between the training and validation set would seriously affect the performance of the radiomics signatures, we proposed a novel approach to split the dataset based on unsupervised K-means clustering algorithm. Firstly, the K-means clustering algorithm was applied to divide radiomics features into 30 sets and the feature nearest to cluster center was considered as the representative one. Then, we randomly split the dataset until there was no significant difference between the training cohort and the validation cohort in 30 representative radiomics features and clinical characteristics (*p*-value > 0.05) (Fig. 2). The 186 patients were divided into a training cohort (*n* = 130) and a validation cohort (*n* = 56) at a ratio of 7:3. The clinical characteristics of included patients in the training and validation cohorts were shown in Table 1.

**Table 1:** The clinical characteristics of the included patients in both the training and validation cohort

Characteristics	Training (***n*** = 130)	Validation (***n*** = 56)	***P***-value
Age, years (mean ± SD)	47.6 ± 13.4	47.8 ± 12.9	0.904
CA125 (mean, range)	552 (5–5000)	526 (10–5000)	0.295
Ki67 (mean ± SD)	28.22 ± 24.09	27.50 ± 22.80	0.467
FIGO (%)			
1	63 (48)	22 (39)	0.553
2	10 (8)	6 (11)	
3	52 (40)	24 (43)	
4	5 (4)	4 (7)	

*P*-value of all characteristics are calculated by one of the independent-samples t-test, the Mann–Whitney U-test or the chi-squared test based on their distribution

### Radiomics signature construction

Before feature selection, up-sampling by repeating random cases was applied to improve the imbalance of the training cohort and we used z-score to normalize the feature matrix. In order to reduce the dimension of features and select more useful features to build the radiomics model, features in the training cohort were divided into several sets according to their categories, such as first-order, shape and texture features. In the radiomics pipeline, combinations of different algorithms were explored to achieve comparative performance. Pearson correlation coefficient (PCC) and feature selection algorithms (feature elimination (RFE), Kruskal-Wallis (KW) test, and Relief) were used to eliminate high correlation features and selection, and classifiers (linear support vector machine (SVM), Logistic Regression (LR), and Random Forest (RF) were applied to predict the prognostic status. The optimal model was selected based on the area under the receiver operating characteristic curve (AUC) in the cross-validation cohort. When the AUC of the model based on the feature subset in the verification set of cross validation is higher than a certain threshold (set to 0.6), all the sub class features used in the model are combined for final modeling. In the final radiomics model, we used PCC and Relief algorithms to select the features used to build the SVM classifier, and 5-fold cross-validation was performed to determine the hyper-parameters of the model in the training cohort. Finally, ten radiomics signatures were built, including four single-sequence signatures and six paired-sequence signatures.

### Radiomic-clinical nomogram construction

The radiomics score (rad-score) was calculated for each patient in the training and validation cohort through the linear combination of the selected features in the radiomics signature. Multivariate logistic regression analysis was performed with the rad-score and clinical characteristics. Based on multivariate logistic analysis, Radiomic-clinical nomogram was constructed in the training cohort to quantitatively predict the prognosis status. We also used clinical characteristics to construct clinical-radiological signature.

### Performance evaluation of the models

We used receiver operating characteristic (ROC) curve and AUC to evaluate the performance of the models. The accuracy (ACC), sensitivity (SEN), specificity (SPE), positive predictive value (PPV) and negative predictive value (NPV) were calculated at the cutoff value according to the Youden index in the training cohort. The calibration curve was performed to evaluate the discrimination of radiomics nomogram. The waterfall plot for distribution of prediction probability and the prognosis status of patients was plotted to verify the predictive ability of the nomogram and decision curve analysis (DCAs), and determined the clinical usefulness and effectiveness of radiomics models by calculating the net benefits at different threshold probabilities in validation cohort.

### Statistical analysis

Statistical analysis was performed with Python (version 3.8). An independent samples t-test or Mann–Whitney U-test was performed to assess the differences in clinical characteristics and radiomic features between the two cohorts, depending on whether they were normal distribution (Kolmogorov–Smirnov test). The difference of categorical variables was assessed with chi-square test. A *p*-value < 0.05 was considered statistically significant. The R software (version 4.0.4, http://www.R-project.org) was performed to plot nomogram, calibration curves and DCAs [29]. The construction of the Radiomics models was implemented on Python using FeAture Explorer Pro (FAEPro, V 4.0.0) [30].

## Results

### Clinical data analysis

In all patient cohorts, according to age, ki67 and FIGO staging, there were significant differences between the good prognosis group and the poor prognosis group (Table 2). The AUC of clinical-radiological signatures was 0.704 (95% CI: 0.619–0.787) in the training cohort and 0.685 (95% CI: 0.545–0.825) in the validation cohort.

**Table 2:** Clinical characteristics of patients in the training and validation cohorts

Characteristics	All cohort (***n*** = 186)		***P***-value	Training cohort (***n*** = 130)		***P***-value	Validation cohort (***n*** = 56)		***P***-value
	Uneventful (***n*** = 124)	Relapse or dead (***n*** = 62)		Uneventful (***n*** = 87)	Relapse or dead (***n*** = 43)		Uneventful (***n*** = 37)	Relapse or dead (***n*** = 19)	
Age (mean ± SD yrs)	44.5 ± 13.8	54.0 ± 9.2	< 0.001	44.3 ± 13.9	54.3 ± 9.0	< 0.001	45.0 ± 13.4	53.5 ± 9.8	0.019
CA125 (mean ± SD Iu/L)	511 ± 884	647 ± 1195	0.472	549 ± 914	557 ± 914	0.384	415 ± 521	740 ± 1275	0.182
Ki67 expression% (mean ± SD)	25.36 ± 23.23	33.31 ± 23.77	0.007	25.98 ± 24.40	32.77 ± 22.78	0.024	23.89 ± 20.16	34.53 ± 25.83	0.102
FIGO stage(%)									
1	65 (52)	20 (33)		48 (55)	15 (35)		17 (46)	5 (27)	
2	9 (7)	7 (11)		5 (6)	5 (12)		4 (11)	2 (10)	
3	48 (39)	28 (45)	0.005	33 (38)	19 (44)	0.029	15 (41)	9 (47)	0.223
4	2 (2)	7 (11)		1 (1)	4 (9)		1 (2)	3 (16)	

### Performance of radiomics signatures for recurrence estimation

**Fig. 3:**
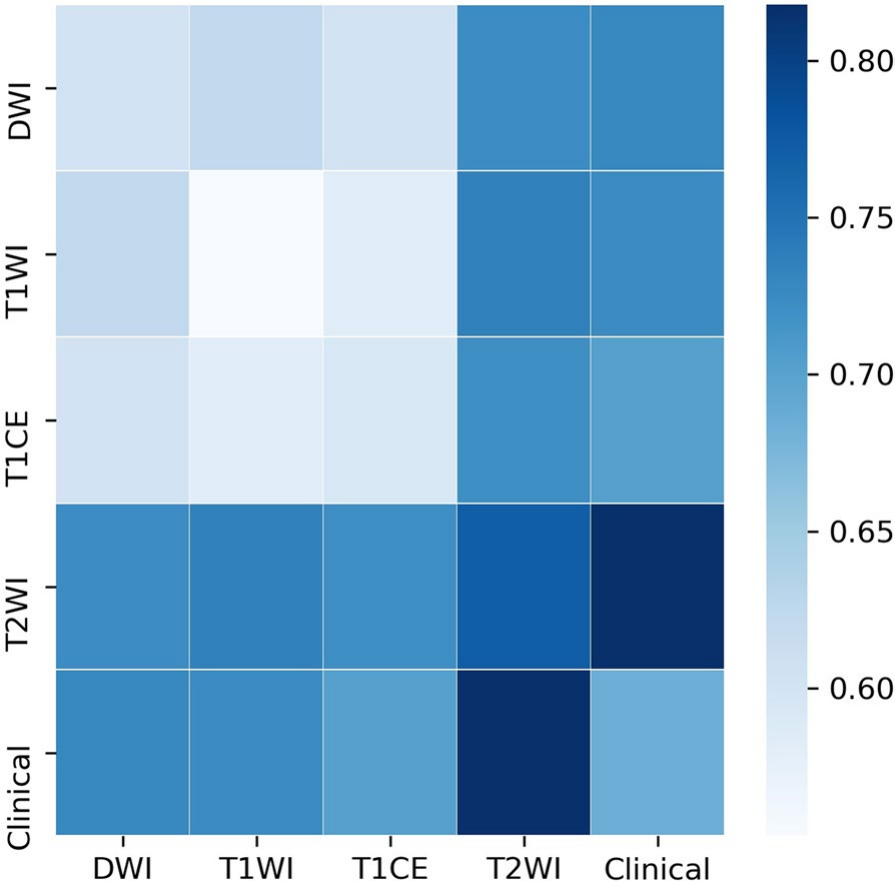
Heat map comparison of the AUC values of radiomics signatures and radiomic-clinical nomogram

The discrimination ability of T2WI, T1CE, and T2WI-T1CE radiomics signatures was evaluated by the ROC curves. T2WI radiomics signature achieved better performance (AUC = 0.771, CI: 0.629–0.894) than T1CE (AUC = 0.593, CI: 0.413–0.770) and T2WI-T1CE (AUC = 0.721, CI: 0.559–0.863) radiomics signatures in the validation cohort. The performance evaluation of all signatures in the validation cohort was listed in Table 3. In the T2WI radiomics signature, 17 radiomics features were selected to build a linear SVM model, and the corresponding coefficients were shown in Supplementary Fig. 1. The selected features in each protocol for prediction model construction and the corresponding contributing coefficients were shown in Supplementary Figs. 2, 3, 4. In brief, the combination of T1WI and T2WI radiomics signatures yielded the highest AUC of 0.736 in the validation cohort (Fig. 3). The comparison of AUC from multi-modal radiomics signatures and the recurrence estimation was summarized in Supplementary Table 2.

**Table 3:** The summaries of performance of different predictive models with radiomics and nomogram in both the training and validation cohort on MR images

Characteristics	Training AUC (95% CI)	Validation AUC (95% CI)	TP	TN	FP	FN	ACC	SEN	SPE	PPV	NPV
Clinical	0.704 (0.619–0.787)	0.685 (0.545–0.825)	17	17	20	2	0.607	0.895	0.459	0.459	0.895
T1WI signature	0.845 (0.771–0.906)	0.553 (0.382–0.736)	10	23	14	9	0.589	0.526	0.622	0.417	0.719
CE T1WI signature	0.837 (0.755–0.910)	0.593 (0.413–0.770)	7	30	7	12	0.661	0.368	0.811	0.500	0.714
DWI signature	0.848 (0.783–0.909)	0.603 (0.441–0.765)	7	15	22	12	0.393	0.368	0.405	0.241	0.556
T2WI signature	0.844 (0.762–0.917)	0.771 (0.629–0.894)	10	31	6	9	0.732	0.526	0.838	0.625	0.775
T1WI nomogram	0.855 (0.794–0.910)	0.724 (0.587–0.865)	14	24	13	5	0.679	0.737	0.649	0.519	0.828
CE T1WI nomogram	0.868 (0.813–0.918)	0.702 (0.557–0.849)	12	24	13	7	0.643	0.632	0.649	0.480	0.774
DWI nomogram	0.767 (0.681–0.850)	0.727 (0.576–0.870)	13	27	10	6	0.714	0.684	0.730	0.565	0.818
T2WI-3D nomogram	0.866 (0.792–0.931)	0.818 (0.691–0.932)	10	33	4	9	0.768	0.526	0.892	0.714	0.786
T2WI-2D nomogram	0.830 (0.765–0.890)	0.720 (0.559–0.873)	13	25	12	6	0.679	0.684	0.676	0.520	0.806

*TP* True positive, *TN* True negative, *FP* False positive, *FN* False negative, *ACC* Accuracy, *SEN* Sensitivity, *SPE* Specitivity, *PPV* Positive predictive value, *NPV* Negative predictive value

### Performance and validation of the radiomics-clinical nomogram

**Fig. 4:**
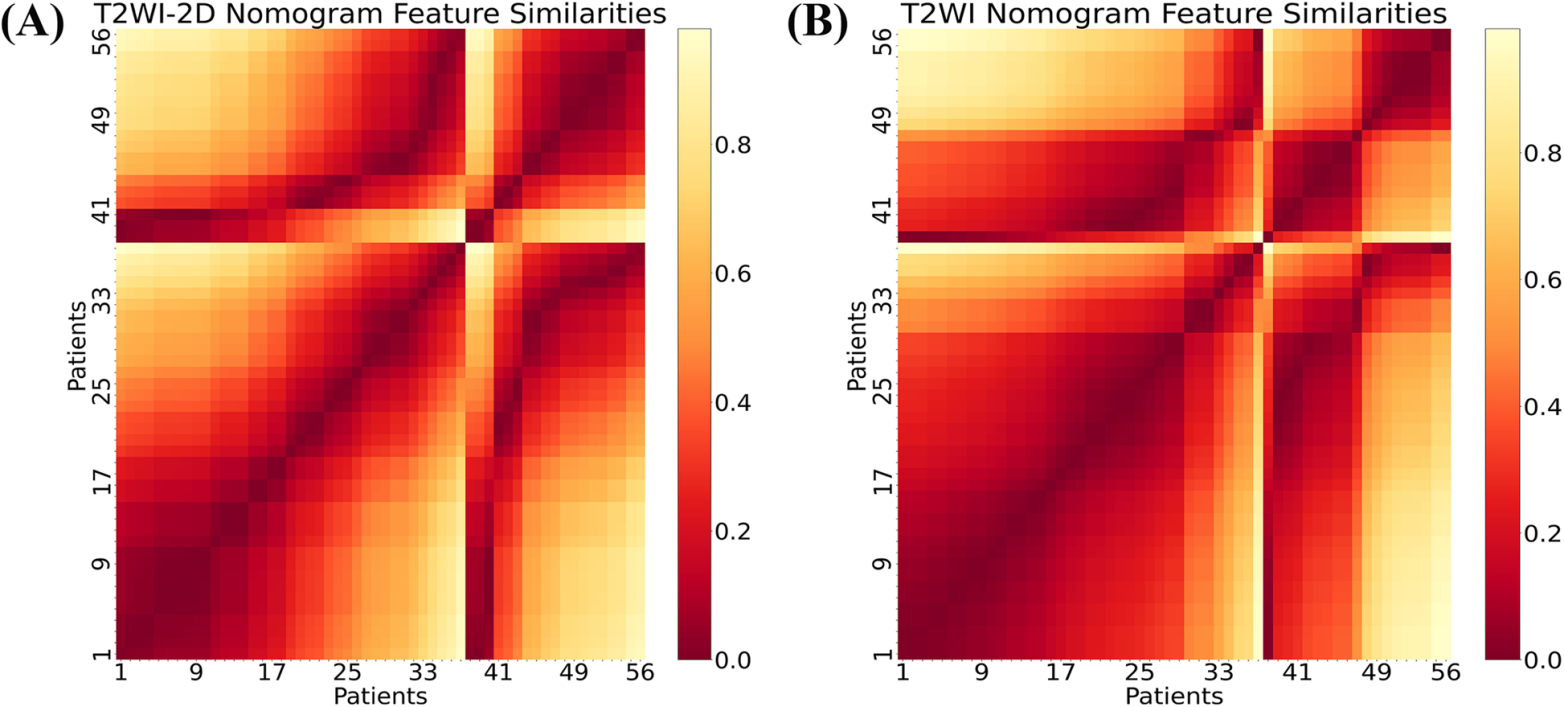
Heat map showing the relative feature similarities of the patients in respect of each other computed by the prediction probability from 2D-T2WI (**A**) or 3D-T2WI radiomics-clinical nomograms (**B**). Prediction probability similarities between two of patients were calculated using Euclidean distance measure. Patients 1–36 were uneventful and patients 37–56 were recurrence or dead. The value close to 0 (red color) means that they had highly similar features

**Fig. 5:**
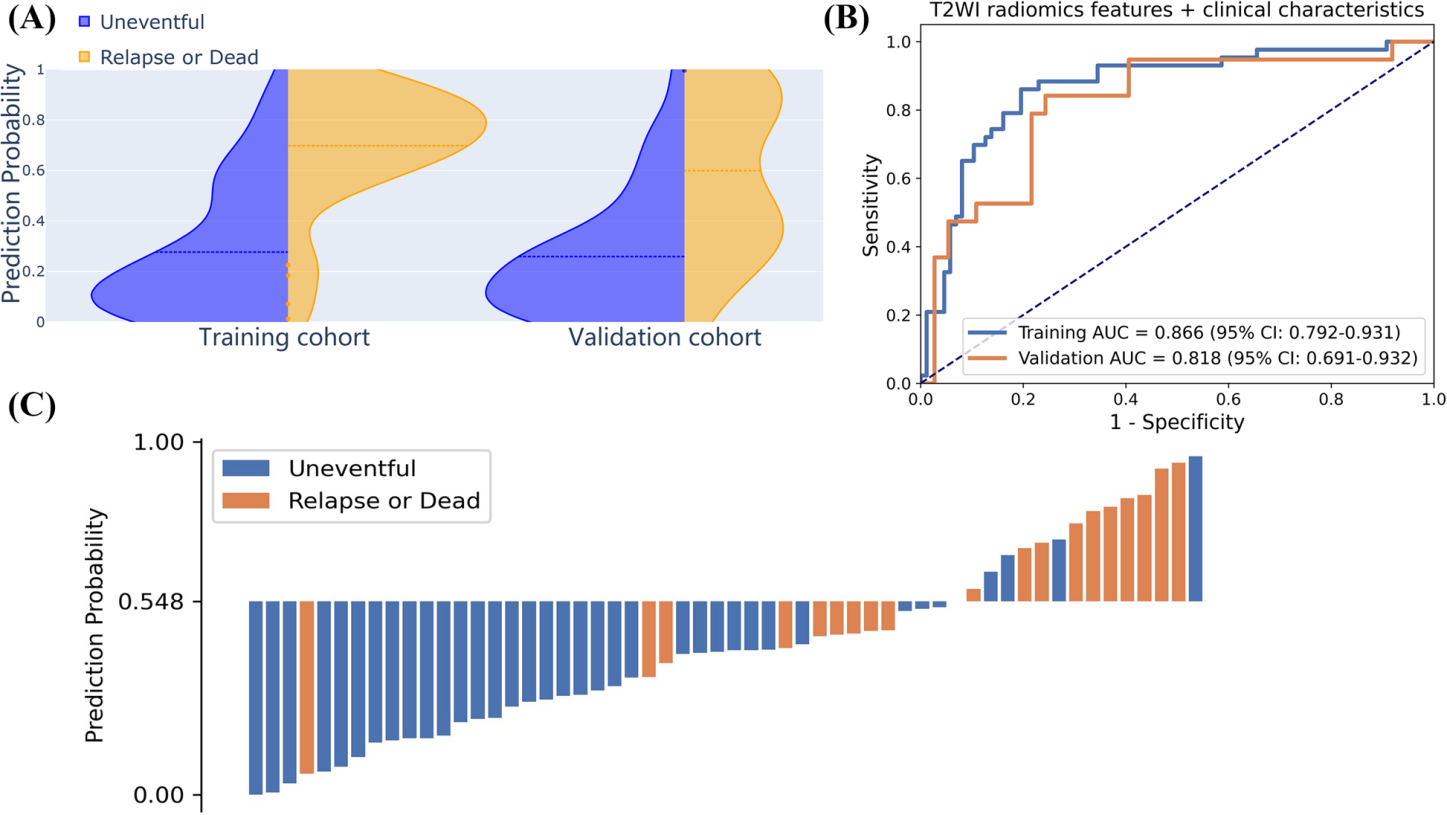
**A** The violin plot for probability density distribution of patients with varying prognosis status in both the training and validation cohort. **B** The ROC curves in the training and validation cohort. **C** The waterfall plot for the distribution of prediction probability of T2WI radiomic-clinical nomogram and the prognosis status of patients in the validation cohort. The cutoff value of 0.548 was defined based on the Youden index in the training cohort

**Fig. 6:**
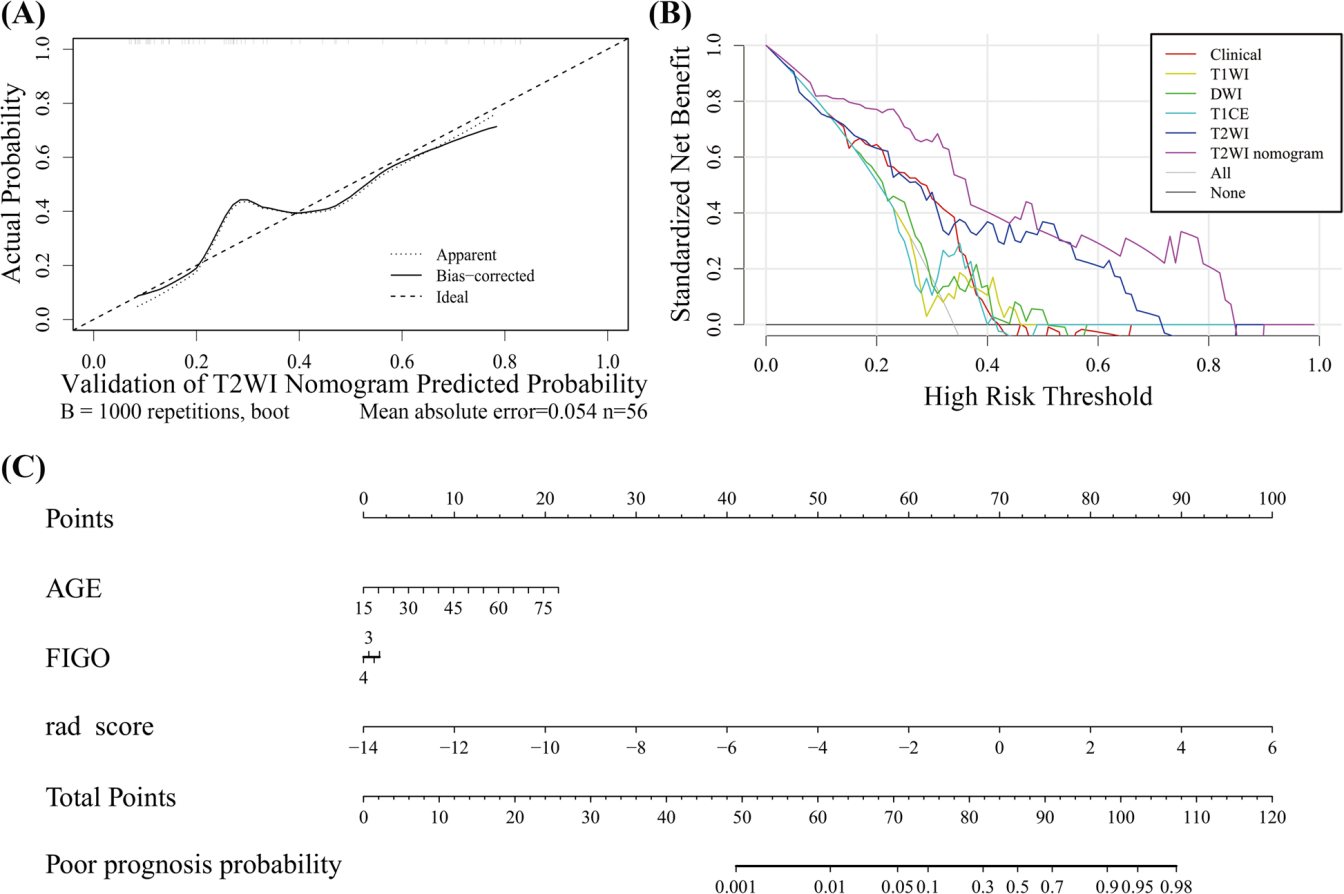
**A** The calibration curve of the T2WI radiomic-clinical nomogram in the validation cohort. The dotted line means the optimal probability prediction model, while the solid line represents the real scenario. An acceptable error occurred because of the imbalanced data. **B** DCA for clinical-radiological signature (red line), T2WI radiomics signature (blue line) and T2WI radiomic-clinical nomogram (purple line). The “All” line is made with the assumption that all patients have poor prognosis. The curve indicates that the net benefit of the nomogram is better than the other models when the threshold is in the range between 0.1 and 0.8. **C** The T2WI radiomic-clinical nomogram incorporated three factors of rad-score, age and FIGO staging

The radiomics-clinical nomogram of each protocol was constructed based on multivariate logistic regression analysis developed by combining rad-score and clinical characteristics. The corresponding evaluation of radiomics-clinical nomogram in both the training and validation cohort was listed in Table 3. The T2WI radiomics-clinical nomogram performed better than other models with an AUC of 0.866 and 0.818, respectively in the training and validation cohort. We also compared the performance of T2WI radiomics-clinical nomogram based on the largest tumor region (two-dimensional, 2D) and the whole tumor region (3D) (Table 3). The prediction probability similarity between two patients was calculated using Euclidean distance measure (Fig. 4). The performance results of 3D T2WI radiomics-clinical nomogram achieved higher similarity than the 2D did for the recurrence prediction. The violin plot and ROC curves of T2WI radiomics-clinical nomogram in the training and validation cohort were shown in Fig. 5A and B. The waterfall plot of the validation cohort with an optimal cutoff value of 0.548 for the distribution of prediction probability of T2WI nomogram and prognostic status was shown in Fig. 5C. Calibration curves with nonsignificant Hosmer-Lemeshow test results (*p*-value = 0.112) and DCAs of radiomics nomogram for prognosis status prediction in the validation cohort also demonstrated favorable performance (Fig. 6).

## Discussion

Ovarian cancer is the most lethal cancer in gynecological tumors. The high heterogeneity of tumor leads to various reactions after treatment, which may influence the prognosis. In this study, we tried to extract preoperative MR-based radiomic signatures and use this noninvasive method to predict prognosis. Our data show that the nomogram combining T2WI-based radiomic signatures with clinical features has high accuracy in predicting the prognosis of selected samples in the training (AUC = 0.866) and validation cohort (AUC = 0.818).

Owing to high soft tissue resolution, MR imaging is always helpful to determine the etiology of adnexal lesions before surgery. Both conventional imaging analysis and imaging-based radiomics studies provide convincing evidence for the classification and prognosis prediction of ovarian masses. Recent MR-based radiomics studies mainly focus on the prediction of ovarian histological subtypes. Radiomics studies can classify EOC patients into binary classifications (Type I and Type II), which is better than conventional MR examination. A recent MR-based radiomics study using multicenter data yielded AUCs of 0.806 and 0.847 in the internal and external validation cohorts for type I and type II EOC discrimination, respectively. The well-known established MR criteria mainly include morphological signs (septa, composition, size, etc.) to discriminate malignant from benign. However, it is difficult to categorize EOC subtypes because of the overlap of the above-mentioned imaging signs.

Compared with the prediction of histological subtypes, the research focusing on the prediction of prognosis is very limited. In a recent study, the authors conducted a retrospective study of 217 patients in one single center and they reported that the radiomic-clinical nomogram showed a favorable predictive ability with an AUC of 0.803, which was used to predict the residual lesion size in ovarian cancer patients undergoing laparotomy [19]. They also concluded that radiomics signature incorporating both CE-T1WI and T2WI features performed better than each sequence alone. In present study, we found that the T2WI-based radiomic signatures achieved better discriminative ability in the prognosis prediction than T1WI, DWI and CE-T1WI alone. Clinical features (age and FIGO staging) are also important clinical characteristics for ovarian cancer categorization [31]. Therefore, the T2WI radiomic-clinical nomogram was constructed by combining the radiomics signature and clinical features to improve the prediction ability.

In respect of dataset split, previous studies mainly focused on the differences in clinical characteristics between the training and validation cohort. However, it is also crucial to ensure a consistent distribution of radiomics features in the two cohorts. Herein, we used the clustering algorithm to select representative features and randomly split dataset until no significant differences were observed in these radiomics features. Most radiomics studies utilized RFE, KW test or Relief algorithms to reduce the feature dimension, and it was usually difficult to obtain the optimal solution due to the high dimension features [16]. In our study, these radiomic features were divided into several sets according to their categories. The subclass features were also used to establish a radiomics-based predictive model. In another study, the authors developed a deep learning method from CT images to establish a CT-based prognostic biomarker for recurrence prediction in high-grade serous ovarian cancer (HGSC) [15]. In this study, they enrolled 245 patients with HGSCs, of which 94 were from two independent centers comprised of the validation cohorts. Their model yielded an AUC of 0.772 to 0.825 for 3-year recurrence prediction. Our present result is a little better than theirs because the nomogram combining T2WI-based radiomic signatures with clinical features has high accuracy in predicting the prognosis of selected samples in training (AUC = 0.866) and validation cohort (AUC = 0.818). However, the advantage of deep learning method is that they can automatically segment the target lesions and are less influenced by the operator himself and his experience. In addition, CT is more widely used in clinical unit to stage the advanced EOC with short scanning time and low expense.

Our study has the following limitations. Firstly, this is a retrospective study of a single center with a relatively small research sample. Larger samples and dependent validation from outer institutions can reasonably explain the results. Secondly, as mentioned above, deep learning technique is gaining more and more attention in medical image analysis. Generating more sophisticated algorithms from a large research sample can improve the performance of preoperative MR to predict the prognosis of EOC patients. Thirdly, owing to the nature of the retrospective study, the treatment methods of all enrolled patients are different, which may also influence the final follow-up results. Prospective design can more clearly clarify the predictive ability of preoperative MR for the outcome of EOC patients after individual treatment.

In conclusion, our current results indicate that MR-based radiomics analysis shows a high degree of accuracy in estimating the prognosis of EOC patients and can help to predict the treatment outcome before treatment. Our future research direction is to better clarify the predictive ability of preoperative MR for EOC patients after individualized treatment through multi-center, large-sample and prospective studies.

## Supplementary Information

**Additional file 1.**

## Data Availability

The authors declare that all data supporting the findings of this study are available within the article.

## Abbreviations

EOC: Epithelial ovarian cancer
HGSC: High-grade serous ovarian cancer
MR: Magnetic resonance
CT: Computer tomography
HIS: Hospital information system
TSE: Turbo spin-echo
T1WI: T1-weighted imaging
T2WI: T2-weighted imaging
DWI: Diffusion weighted imaging
VOI: Volume of interest
PCC: Pearson correlation coefficient
AUC: Area under curve
ROC: Receiver operating characteristic
PPV: Positive predictive value
NPV: Negative predictive value
2D: Two-dimensional
3D: Three-dimensional
