# Supplementary material for: MR-based radiomics-clinical nomogram in epithelial ovarian tumor prognosis prediction: tumor body texture analysis across various acquisition protocols
Source: J Ovarian Res. 2022 Jan 12;15:6. doi: 10.1186/s13048-021-00941-7 (PMC8753904; doi:10.1186/s13048-021-00941-7)
Supplement: Supplementary file 1 — Additional file 1. [file 13048_2021_941_MOESM1_ESM.docx]

**Supplementary files**

**Figure 1.**

**
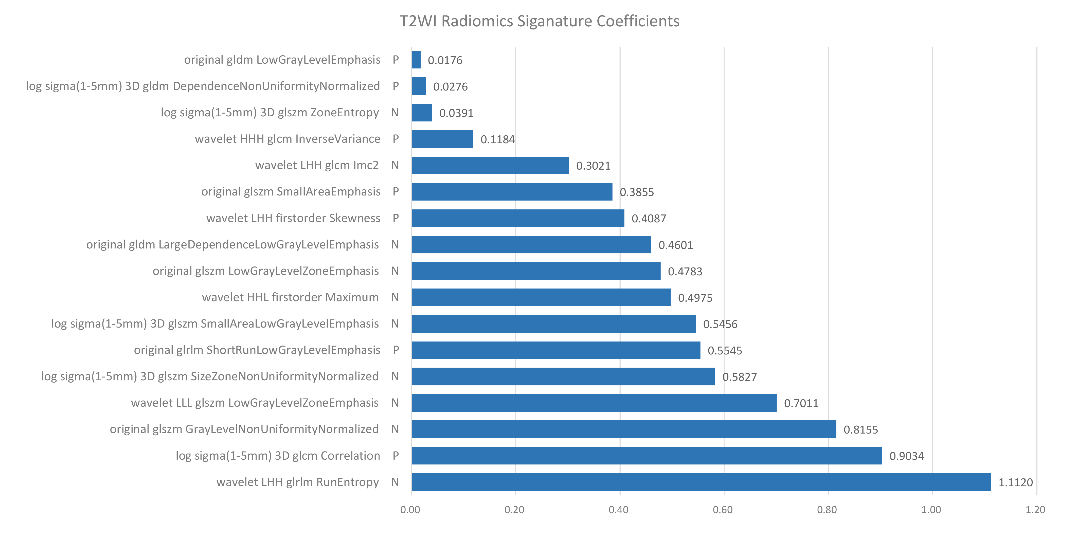
**

**Figure 2**

**
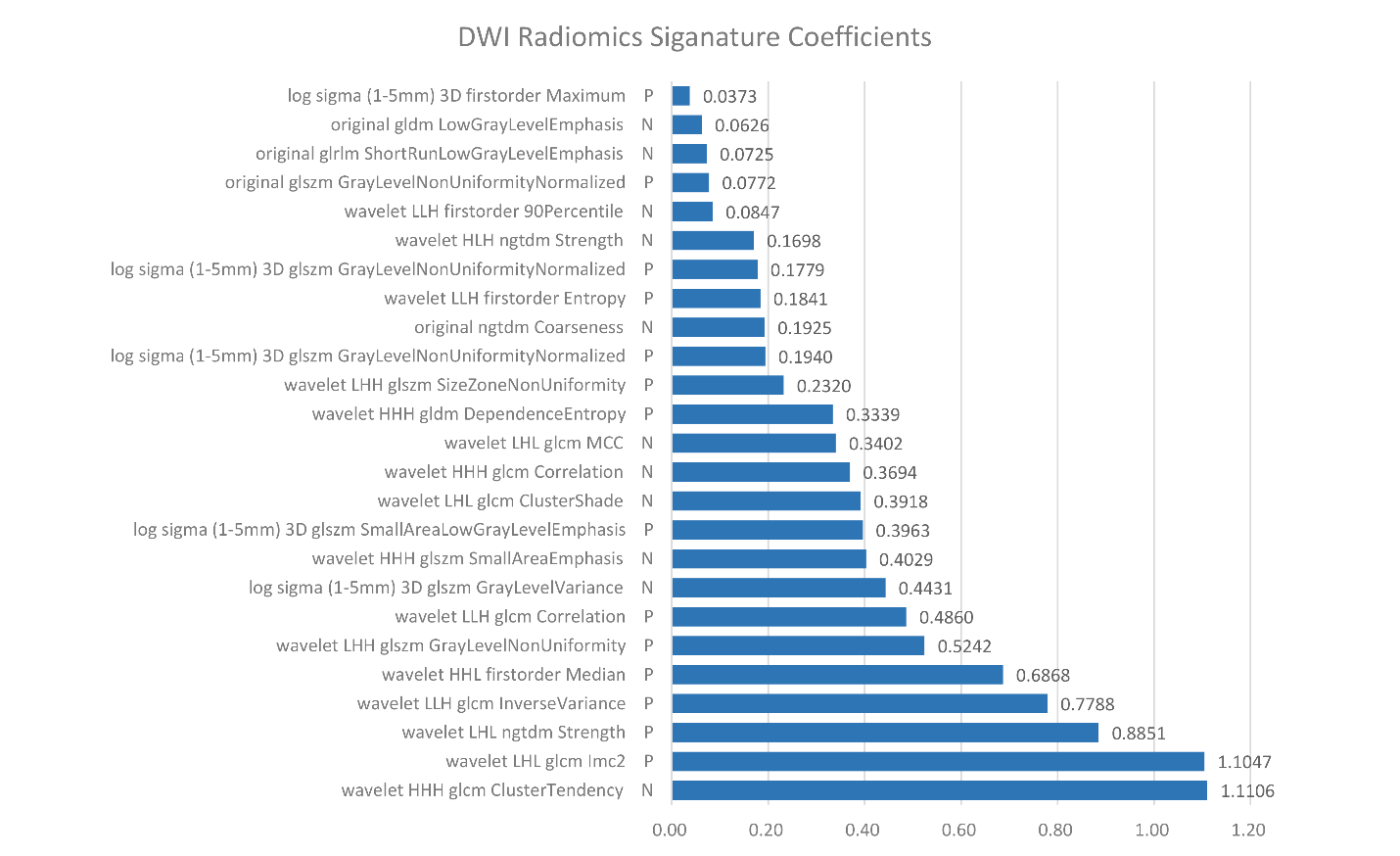
**

**Figure 3.**

**
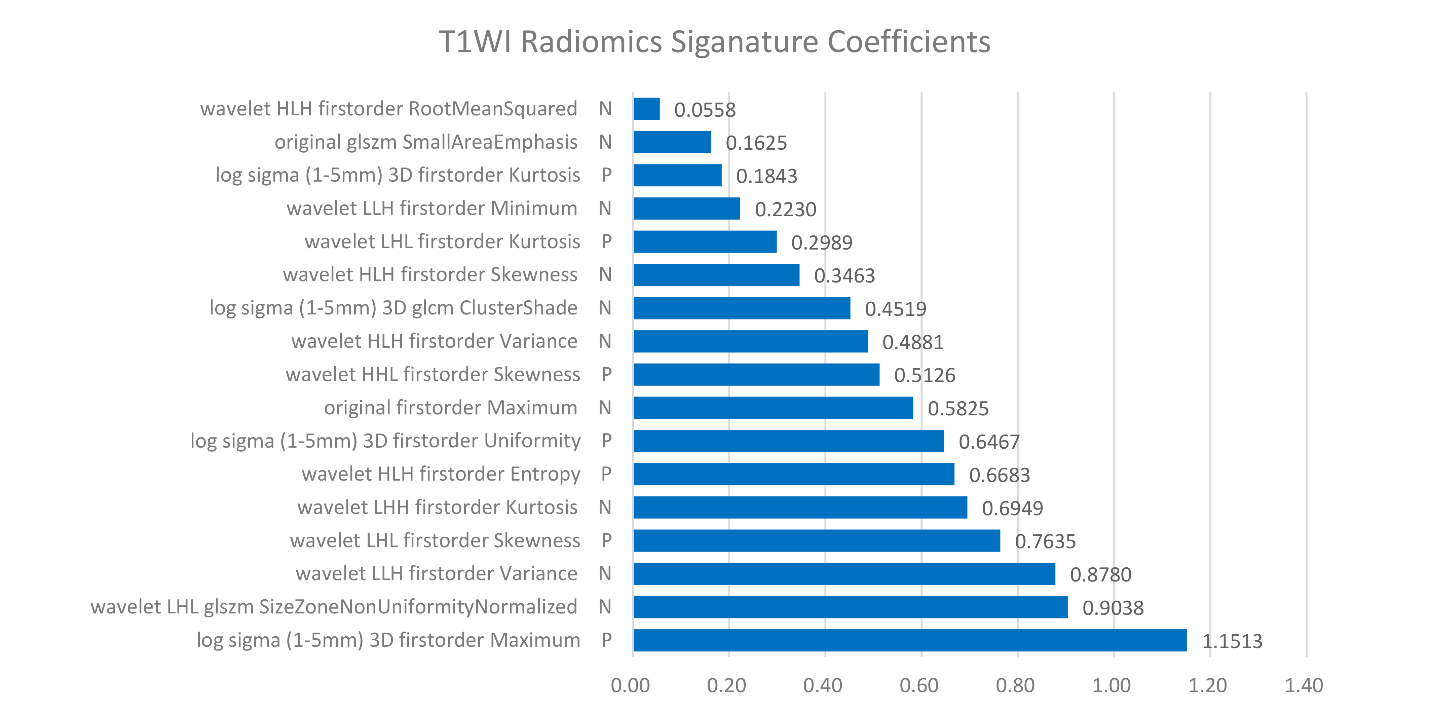
**

**Figure 4.**

**
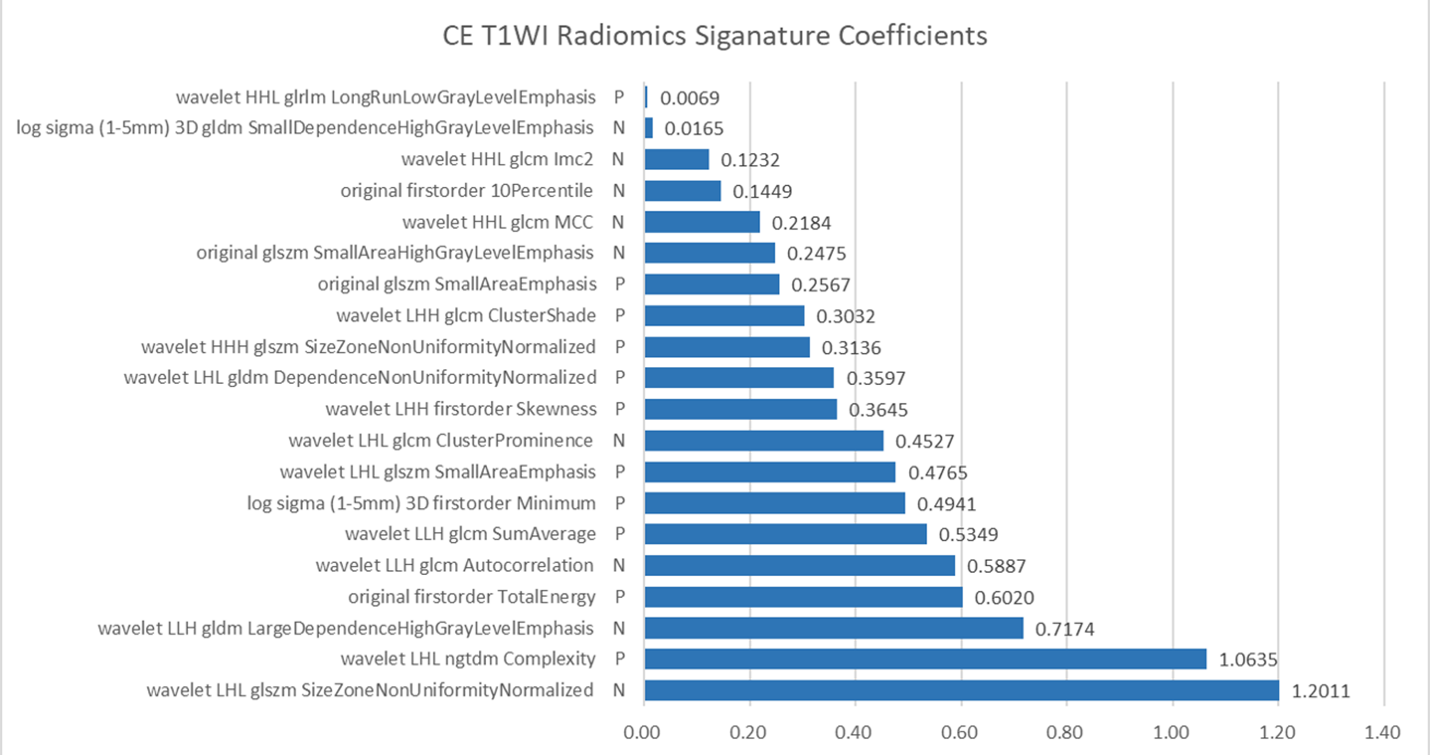
**

**Table**

| **Table 1**. Details of parameters for 1.5 Tesla MRI imaging protocols | | | | | |
| --- | --- | --- | --- | --- | --- |
| Parameters | T1WI | T2WI | FS-T2WI | DWI(*b* =0, 800mm^2^/s) | Contrast-enhanced MRI |
| Repetition / echo time (msec) | 550/10 | 4000/83 | 8000/83 | 2800/81 | 4.89/2.38 |
| Echo trains per slice | 44 | 19 | 19 |  |  |
| Sequence | TSE | TSE | TSE | EP2D | VIBE |
| Bandwidth (Hz) | 178 | 260 Hz | 260 | 1250 Hz | 400 Hz |
| Thickness(mm) | 4 | 4 | 4 | 5 | 3 |
| Gap(mm) | 1.2mm | 1.2mm | 1.2mm | 1.5mm | 0.9mm |
| Field of view (mm) | 350 | 350 | 350 | 300 | 380 |
| Voxel size(mm) | 1.5×1.1×4.0 | 1.1×1.1×4.0 | 1.4×1.4×4.0 | 2.7×1.9×5.0 | 1.7×1.2×3.0 |
| Flip angle (degrees) | 150 | 144 | 150 |  | 10 |

**Table 2.** The performance of paired-sequence radiomics signatures in both the training and validation cohort

| **Characteristics** | **Training AUC**  **(95% CI)** | **Validation AUC**  **(95% CI)** | **TP** | **TN** | **FP** | **FN** | **ACC** | **SEN** | **SPE** | **PPV** | **NPV** |
| --- | --- | --- | --- | --- | --- | --- | --- | --- | --- | --- | --- |
| DWI+CE T1WI  signature | 0.780  (0.706-0.848) | 0.602  (0.449-0.752) | 11 | 20 | 17 | 8 | 0.554 | 0.579 | 0.541 | 0.393 | 0.714 |
| DWI+T1WI  signature | 0.807  (0.740-0.868) | 0.622  (0.445-0.794) | 10 | 26 | 11 | 9 | 0.643 | 0.526 | 0.703 | 0.476 | 0.743 |
| DWI+T2WI  signature | 0.929  (0.882-0.968) | 0.723  (0.570-0.856) | 13 | 26 | 11 | 6 | 0.696 | 0.684 | 0.703 | 0.542 | 0.813 |
| T1WI+CE T1WI  signature | 0.904  (0.853-0.948) | 0.583  (0.427-0.733) | 3 | 32 | 5 | 16 | 0.625 | 0.158 | 0.865 | 0.375 | 0.667 |
| T1WI+T2WI  signature | 0.823  (0.758-0.887) | 0.736  (0.572-0.887) | 8 | 34 | 3 | 11 | 0.750 | 0.421 | 0.919 | 0.727 | 0.756 |
| T2WI+CE T1WI  signature | 0.908  (0.864-0.948) | 0.721  (0.569-0.870) | 10 | 32 | 5 | 9 | 0.750 | 0.526 | 0.865 | 0.667 | 0.780 |
